# Travelling beyond time: shared brain system for self-projection in the temporal, political and moral domains

**DOI:** 10.1098/rstb.2023.0414

**Published:** 2024-09-16

**Authors:** Amnon Dafni-Merom, Rotem Monsa, Meitar Benbaji, Adi Klein, Shahar Arzy

**Affiliations:** ^1^ Neuropsychiatry Laboratory, Department of Medical Neurosciences, Faculty of Medicine, Hebrew University of Jerusalem, Jerusalem 9190501, Israel; ^2^ Department of Neurology, Hadassah Hebrew University Medical School, Jerusalem, Israel; ^3^ Department of Cognitive and Brain Sciences, Hebrew University of Jerusalem, Jerusalem 9190501, Israel

**Keywords:** mental time travel, self-projection, self-reference, fMRI, politics, morality

## Abstract

Mental time travel (MTT), a cornerstone of human cognition, enables individuals to mentally project themselves into their past or future. It was shown that this self-projection may extend beyond the temporal domain to the spatial and social domains. What about higher cognitive domains? Twenty-eight participants underwent functional magnetic resonance imaging (fMRI) while self-projecting to different political, moral and temporal perspectives. For each domain, participants were asked to judge their relationship to various people (politicians, moral figures, personal acquaintances) from their actual or projected self-location. Findings showed slower, less accurate responses during self-projection across all domains. fMRI analysis revealed self-projection elicited brain activity at the precuneus, medial and dorsolateral prefrontal cortex, temporoparietal junction and anterior insula, bilaterally and right lateral temporal cortex. Notably, 23.5% of active voxels responded to all three domains and 27% to two domains, suggesting a shared brain system for self-projection. For ordinality judgement (self-reference), 52.5% of active voxels corresponded to the temporal domain specifically. Self-projection activity overlapped mostly with the frontoparietal control network, followed by the default mode network, while self-reference showed a reversed pattern, demonstrating MTT’s implication in spontaneous brain activity. MTT may thus be regarded as a ‘mental-experiential travel’, with self-projection as a domain-general construct and self-reference related mostly to time.

This article is part of the theme issue ‘Elements of episodic memory: lessons from 40 years of research’.

## 1. Introduction

Humans can mentally disengage from the here and now and project themselves back in time or into future events, a capacity termed ‘mental time travel’ (MTT) [1–4]. In doing so, individuals can relive past experiences and anticipate future occurrences, creating a sense of continuity across time that is central to human consciousness [5]. Further, the ability to mentally travel to different points in time, while maintaining a stable sense of self, is crucial in the formation of a ‘narrative-’ [6] or ‘extended-self’ [7], which is subjectively aware of its past and future.

Self-projection is a major cognitive construct that is an essential part of MTT, signifying the ability of individuals to shift perspective from the immediate present to specific alternative perspectives [8,9]. It has been suggested that a shared brain network supports diverse forms of self-projection, including prospective future thinking, remembering the past, theory of mind (ToM) and navigation [10]. In this sense, one line of research applied a mechanistic approach, originally focusing on self-projection to specific time points along the mental timeline and the concurrent chronometrical computations [9,11–13]. Further research compared the underlying neuroanatomy of MTT and mental space navigation, that is the human ability to imagine themselves in different spatial self-locations [14,15]. Self-projection in time and space was found to be processed by distinct dimension-specific cortical networks, overlapping in the right inferior parietal lobe, precuneus and superior frontal cortex [15]. More recently, this approach was also applied to study self-projection in the social domain [16]. Similarly, it was found that self-projection in this domain involved the activation of the medial parietal cortex, temporoparietal junction (TPJ), medial prefrontal cortex (mPFC) and superior frontal cortex. These neural underpinnings largely overlap with the so-called *core network* (not to be confused with the default core network referred to in this paper as DMN-A [17,18]), a network that fundamentally supports the (re)construction and simulation of past and future episodes [19–22]. Further, an intracranial electroencephalogram study showed that temporal self-projection activates brain regions within the lateral temporal lobe [23], also a hub of the core network. Taken together, these results suggest that the core network may not only support temporal self-projection but may also play an expanded role, flexibly enabling the mental representation of a continuous axis and the projection of oneself to different points on this axis in different domains [24,25].

Imagining oneself across different temporal points necessitates a shift in mental perspective in relation to one’s environment. Self-reference, another important cognitive construct necessary for MTT, depicts this ability to refer to other events, places or people from a newly imagined (or actual) self-location ([5,12,24–27]; for the self-reference effect in memory see [28–31]). In the MTT task, participants are asked to project themselves to specific self-locations in time (past, present or future) and then assess whether events occurred before (relative-past) or after (relative-future) their self-location in time [8,9,11–13,32]. Previous studies indicate reaction times and success rates are comparable in past and future self-locations but are slower and less accurate than in the present self-location, known as the ‘self-projection effect’ [8,9,12,15]. They also show that the same event can be perceived differently based on the imagined self-location, highlighting imagination’s impact on the cognitive processing of events. Namely, relative-future events have been reported to be processed more quickly and accurately than relative-past events, regardless of the self-location [8,9]. Hence, the experiential self plays a crucial role in forming and interpreting events on a mental timeline, a role that may be extended beyond the mental timeline to mental space [14,15,33]. The default mode network (DMN) is a brain network known to be related to self-referential internal thinking, including mind-wandering, ToM and autobiographical memory [34,35]. Accordingly, self-reference in another cognitive domain, the social domain, was found in a previous study to significantly overlap the DMN [16].

Given the implication of self-projection and self-reference not only in MTT but also the spatial and social domains [10,15,16], here we inquire whether this may reflect underlying domain-general brain systems for these core functions [5]. We hypothesize that if indeed this is the case, these domain-general systems should process self-projection and self-reference in other domains of cognition. As the spatial, temporal and social domains are developed early in life [36–41], our focus here was on cognitive domains that emerge later in life, such as politics and morality. Thus, we ask whether self-projection to different political and/or moral perspectives and self-reference to the corresponding environments are supported by similar neurocognitive systems as MTT.

## 2. Material and methods

### (a) Participants

Twenty-eight healthy volunteers (17 females, mean age: 25.4 ± 2.2 years old) participated in the study. Beyond these 28 individuals, seven additional volunteers were enrolled but excluded owing to excessive head movements during image acquisition (*n* = 6) and technical issues during functional magnetic resonance imaging (fMRI) data acquisition (scanner cooling malfunction during the scan, *n* = 1). All participants were native Hebrew speakers, right-handed, had normal or corrected-to-normal vision and had no history of neurological or psychiatric disorders. All participants provided written informed consent, and the study was approved by the Ethical Committee of Hadassah Medical Center in accordance with the Declaration of Helsinki [42].

### (b) Stimuli and procedure

At least a week before the experiment, participants were asked to provide names of people and specify either their relative political views (much more right-wing, more right-wing, slightly more right-wing, slightly more left-wing, more left-wing), moral standpoint (much more immoral, more immoral, slightly more immoral, slightly more moral, more moral) or wedding dates (got married 6–10 years ago, got married 0–5 years ago, expected to marry within 0–5 years, expected to marry within 6–10 years, expected to marry within 11–15 years). This enabled the use of the same kind of stimuli across all domains and gave rise to a set of 30 names for each of the three domains studied in the experiment (political, moral and temporal), categorized and evenly distributed according to the cognitive distance from each participant’s actual political views, moral standpoint or temporal self-location. For the temporal domain, participants provided names of personal acquaintances, while for the political and moral domains, participants provided names of political and moral figures, respectively. This enabled participants to yield stimuli from the far ends of the political and moral spectrums.

**Figure 1.**
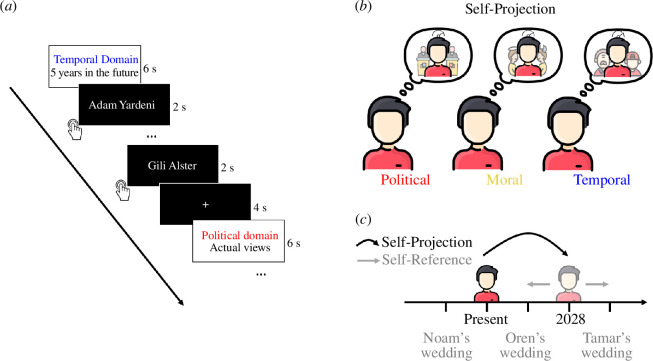
Experimental paradigm. (*a*) Each block began by presenting the domain (political, moral or temporal) and self-location (actual or projected) for 6 s (white screen). This instruction screen was followed by eight consecutive trials, in which participants were presented with the names of persons (black screens). For each domain, participants indicated the ordinality of the person with respect to their self-location (left-wing/right-wing, immoral/moral or got married/expected to marry) using left–right button responses. (*b*) Participants performed the task for each domain from their actual or projected self-location, as indicated by the instruction screen. In the self-projected condition, participants were asked to imagine themselves as leaning more to the right politically, more immoral, or 5 years in the future and to perform the task accordingly from this imagined self-location. (*c*) An example from the temporal domain in which participants were asked to project themselves to an imagined self-location in the future. From this imagined self-location, or the present one, they were asked to judge whether the event has already happened or is yet to happen, relative to their self-location in time (for further details see §2b, ’Stimuli and procedure’).

In the experimental task, an adaptation of the MTT task [8,9,11–13,15,43], participants were presented with names of people and were asked to determine for each whether the person is more left- or right-wing (for the political domain), more moral or immoral (for the moral domain), and got married or expected to get married (for the temporal domain) (figure 1*a*). Notably, to achieve uniformity across domains, stimuli consisted of people’s names, thereby tapping into the social cognition level to a certain degree. However, participants were not instructed to focus on their social relationships to these stimuli, rather the names served as proxies to events and political and moral views. Participants were asked to perform the task either with respect to their actual political, moral or temporal self-location or with respect to an imagined, ‘projected’ self-location (more right-wing political view, more immoral outlook, or themselves 5 years in the future, respectively; figure 1*b*). For example, in the temporal domain, a participant anticipating that their brother might get married within the next 5 years may indicate ‘will get married’ in the actual self-location condition, but indicate ‘has got married’ in the projected self-location condition (figure 1*c*). Therefore, in this experimental paradigm, self-projection refers to one’s ability to travel along three different mental lines—temporal, political and moral—and localize oneself in a different imaginary point. Self-reference is the ability to refer to these events, political views or moral standpoints from the newly imagined or actual, self-location. Importantly, stimuli were presented to the participants only after mentally travelling (or self-projecting) in the relevant conditions. Participants also performed a lexical control condition, in which they saw the same names as in the experimental conditions, but here were asked to indicate whether the name contained a specific letter.

On the day of the experiment, participants were shown written instructions concerning the experimental task and performed a short training session outside the scanner. This allowed the participants to familiarize themselves with the response-key layout and clarify any issue regarding the task instructions. Responses were recorded by pressing the corresponding (left or right) buttons. Stimuli were presented in a randomized block design. Each block started with an instruction screen (6 s) indicating the relevant domain and whether the task should be performed from the actual or projected self-location. This was followed by the consecutive presentation of eight stimuli, each for 2 s (figure 1*a*). A fixation cross was presented following each experimental block (4 s). Participants were instructed to respond accurately but as quickly as possible. The experiment consisted of six experimental runs for each participant, each containing 28 blocks in a randomized order (24 experimental blocks, counterbalanced for cognitive distance and self-projection; four lexical control blocks). Stimuli were presented using the Presentation software (v. 18.3, Neurobehavioral Systems, Inc., Berkeley, CA, www.neurobs.com).

### (c) Analysis of behavioural data

To investigate the behavioural effect of self-projection and self-reference, a two-by-two repeated measures analysis of variance (ANOVA) was performed for each domain separately on participants average response times (RT) and success rates (SR), with factors for self-location (actual or projected) and self-reference (temporal domain: relative-past or relative-future; moral domain: relative-immoral or relative-moral; political domain: relative-left-wing or relative-right-wing). The *p*-values were adjusted using the Bonferroni method to account for multiple comparisons. Furthermore, a repeated measures ANOVA was conducted comparing the RTs of the actual blocks of the three studied domains and the lexical control condition. Behavioural data analysis was performed using the SPSS software (IBM SPSS Statistics, v. 29.0.2.0 Armonk, NY: IBMCorp.).

### (d) Magnetic resonance imaging acquisition

Subjects were scanned in a 3T Siemens Skyra MRI (Siemens, Erlangen, Germany) at the Edmond and Lily Safra Center (ELSC) neuroimaging unit. Blood oxygenation level-dependent (BOLD) contrast was obtained with a gradient-echo, echo-planar imaging sequence (time to repetition (TR) = 2000 ms; time to echo (TE) = 32.4 ms; flip angle = 78°; field of view = 192 mm; matrix size = 96 × 96; functional voxel size = 2 × 2 × 2 mm; 72 slices, multi-band acceleration factor = 4, interleaved acquisition order = 368 TRs per run). In addition, a resting-state scan of 120 TRs with identical parameters was performed. Finally, T1-weighted high-resolution (1 × 1 × 1 mm, 160 slices) anatomical images were acquired for each subject using the MPRAGE protocol (TR = 2300 ms; TE = 2.98 ms; flip angle = 9°; field of view = 256 mm).

### (e) Magnetic resonance imaging preprocessing

fMRI data were processed and analysed using the BrainVoyager 20.6 software package (R. Goebel, Brain Innovation, Maastricht, The Netherlands), Neuroelf v. 1.1 (www.neuroelf.net) and in-house MATLAB scripts (MathWorks). Preprocessing of functional scans included slice timing correction (cubic spline interpolation), three-dimensional motion correction by realignment to the first run image (trilinear detection and sinc interpolation), high-pass filtering (up to two cycles), smoothing (Full-width at half-maximum = 4 mm), exclusion of voxels below intensity values of 100 and coregistration to the anatomical T1 images. Runs with maximal motion above a single voxel size (2 mm) in any direction were removed from further analyses. Anatomical brain images were corrected for signal inhomogeneity and skull-stripped. Subsequently, all images were normalized to Montreal Neurological Institute space (functional resolution = 2 × 2 × 2 mm, trilinear interpolation).

### (f) Functional magnetic resonance imaging analyses

#### (i) General linear model

To assess the selective activations elicited by different experimental conditions, we applied a general linear model (GLM) analysis [44]. The model predictors corresponded to the experimental conditions (domain: temporal, moral or political; self-location: actual or projected and lexical control). Each modelled predictor included all experimental blocks at one condition, where each block was modelled as a boxcar function encompassing the seven conditions. Predictors were convolved with a canonical haemodynamic response function, and the model was fitted to the BOLD time course at each voxel. Six motion parameters (three translations, three rotations) were added to the GLM to eliminate motion-related noise. In addition, white matter and cerebrospinal fluid masks were manually extracted in BrainVoyager for each participant (intensity > 150 for the white matter mask and intensity < 10 with a bounding box around the lateral ventricles for CSF) and the average signals from these masks were added to the GLM to eliminate potential noise sources. Data were corrected for serial correlations using the AR(2) model and transformed into units of per cent signal change. Subsequently, a random-effects analysis was performed across all subjects to obtain group-level beta values for each predictor.

### (g) Identification of domain-specific and domain-general activity

To identify regions that respond to self-projection for each specific domain, we used a balanced contrast between the projected blocks of each specific domain (temporal, moral, political) and the average of the projected blocks of the other two domains. To identify activations underlying self-reference in each specific domain, we used a balanced contrast between the actual blocks of each specific domain and the average of the other two domains. These contrasts identified regions responding specifically to self-projection or self-reference in only one domain, respectively. To identify the full extent of activation for self-projection in each domain, we contrasted the projected and actual blocks of each domain. Similarly, we contrasted the actual blocks of each domain with the lexical control task to identify the full extent of self-reference for each domain. These last two contrasts enabled the detection of overlap of activations between multiple domains. For self-reference contrasts, the past/future, left-wing/right-wing and immoral/moral trials within blocks were collapsed. Clusters with a clusterwise false discovery rate (FDR)-corrected *p*-value of less than 0.05 were retained. Clusters below a cluster size threshold of 25 voxels were excluded from the abovementioned contrasts. Cluster coordinate tables and region labels were generated using AtlasReader [45] (https://github.com/miykael/atlasreader).

### (h) Overlap between domains

To identify overlap between regions responding to self-projection, activation clusters were isolated from the contrast between the projected and actual blocks of each domain, as described above (§2g, ‘Identification of domain-specific and domain-general activity’). To assess the overlap between the domains, we calculated the percentage of voxels significantly active in two or three domains separately, compared with the total number of active voxels. Percentages of overlapping voxels were averaged across subjects. This same analysis was performed on the contrast between actual blocks and the control task for each domain, to identify overlap between regions responding to self-reference.

### (i) Comparison of task-related activations to large-scale resting-state networks

Next, we aimed to compare the resulting brain activity to known brain networks. A previously published whole-brain parcellation into seven large-scale brain networks was used as a template for the location of resting-state networks [17] (https://surfer.nmr.mgh.harvard.edu/fswiki/CorticalParcellation_Yeo2011). To characterize the involvement of each resting-state network in self-projection and self-reference for each domain (temporal, moral and political), we calculated the Jaccard index (intersection over union) between the group-level GLM results (voxels with overlapping activations in at least two domains) and the cortical parcellation to seven large-scale brain networks. In a second step, we calculated the overlap with 17 network parcellations available at the above resource [17] to determine the overlap with three subdivisions of the DMN (DMN-A, DMN-B and DMN-C) and two subdivisions of the frontoparietal control network (FPCN-A and FPCN-B). FPCN subdivisions are based on a fractionation according to functional connectivity patterns, where FPCN-A is connected to the DMN and is involved in regulating introspective processes, while FPCN-B is connected to the dorsal attention network and is involved in regulating perceptual attention [46] (https://github.com/adelavega/fpcn_fractionation). For DMN subdivisions, DMN-A (also dubbed as the ‘midline core network’) correlates with self-related processes and social and mnemonic processes shared by the other two subdivisions, DMN-B with mentalizing, social cognition, story comprehension and semantic processing, and DMN-C with past and future autobiographical thinking, episodic memory and contextual retrieval [18]. To assess preference for specific subdivisions of the DMN and FPCN, *χ* ^2^ goodness-of-fit was calculated, comparing the distribution of the self-projection and self-reference group-level results within the subdivisions of the DMN and the FPCN with the theoretical expected distribution of the results given the proportions of the subdivisions. Next, to assess whether the observed differences in overlapping voxel proportions are significant, pairwise *χ* ^2^-tests were conducted to compare the proportions of overlapping voxels between each pair of subdivisions. Specifically, for each pair, a 2 × 2 contingency table was created, and a *χ* ^2^-test was applied. Volume results were converted to surface representations and displayed using Connectome Workbench software [47].

To test whether the DMN and FPCN are co-activated or decoupled during self-projection, we performed a task-based functional connectivity analysis using in-house Python scripts (Python 3.9) according to Cole *et al*. [48]. First, confounds (white matter, CSF and six motion parameters) were regressed out of the preprocessed fMRI signal. Next, the posterior cingulate cortex (PCC; a major hub of the DMN-A) and FPCN-A were defined as regions of interest (ROIs), following the results of the previous analysis. For each ROI, seeds were created by averaging the activity time course of all associated voxels. Subsequently, the relevant task conditions (projected and actual) were separately regressed from the signal. Time points with null values for all relevant task regressors were omitted. Finally, Pearson’s correlation was applied between the time points of the ROIs and *r*-values were converted to *z*-values using Fisher’s transformation. This resulted in two connectivity values for each subject, for the projected and the actual conditions. Two-tailed paired *t*-tests were performed to examine the effect of self-projection on functional connectivity between the ROIs. We report the *r*-values corresponding to the transformed *t*-values. Correction for multiple comparisons was carried out using the FDR approach [49]. A similar analysis was conducted for self-reference, comparing the functional connectivity between the actual versus control conditions in the same ROIs.

## 3. Results

### (a) Better performance for actual compared with projected self-location across domains

**Figure 2.**
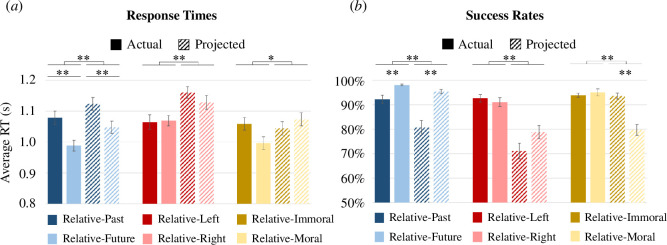
Behavioural results. Participants had (*a*) longer average RTs, and (*b*) lower SRs for the self-projection conditions (dashed bars) compared with the actual (no projection) conditions (solid bars) in all three domains. Participants had (*a*) longer average RTs for the relative-past condition (dark blue bars) compared with the relative-future condition (light blue bars), and (*b*) lower SRs for the relative-past condition compared with relative-future condition, as well as higher SRs for the relative-immoral condition (dark gold bars) compared with relative-moral condition (light yellow bars). Error bars refer to the standard error of the mean (s.e.m.). An asterisk denotes *p* < 0.05 and a double asterisk denotes *p* < 0.001. The *p*-values are Bonferroni-corrected to account for multiple comparisons.

Previous studies have shown that self-projection in time results in slower RTs and lower SRs, as disengaging from the here and now entails a behavioural cost [9,15,33]. As expected, our behavioural analysis indicates that self-projection in time leads to slower RTs (*F*_1, 27_ = 36.045, *p* < 0.001, $\eta_{P}^{2}$ = 0.572; figure 2*a*) and lower SRs (*F*_1, 27_ = 18.172, *p* < 0.001, $\eta_{P}^{2}$ = 0.402; figure 2*b*). Similarly, self-projection in the political and moral domains resulted in slower RTs (*F*_1, 27_ = 19.835, *p* < 0.001, $\eta_{P}^{2}$ = 0.424 and *F*_1, 27_ = 9.266, *p* = 0.005, $\eta_{P}^{2}$ = 0.256, respectively; figure 2*a*) and lower SRs *F*_1, 27_ = 42.623, *p* < 0.001, $\eta_{P}^{2}$ = 0.612 and *F*_1, 27_ = 45.097, *p* < 0.001, $\eta_{P}^{2}$ = 0.626, respectively; figure 2*b*). Notably, the behavioural costs of self-projection in the temporal, political and moral domains were not significantly different for RTs (repeated measures ANOVA, *F*_2,26_ = 1.793, *p* = 0.18) or SRs (repeated measures ANOVA, *F*_2,26_ = 2.058, *p* = 0.14). These findings allow us to rule out trivial effects of task difficulty (or cognitive load) when comparing the neural correlates of self-projection in these domains [15]. Taken together, these results indicate that participants have indeed performed the mental operation of self-projection in each domain, as manifested by the additional cognitive load.

For the temporal domain, participants responded significantly faster (*F*_1, 27_ = 53.774, *p* < 0.001, $\eta_{P}^{2}$ = 0.666; figure 2*a*) and with higher SRs (*F*_1, 27_ = 30.973, *p* < 0.001, $\eta_{P}^{2}$ = 0.534; figure 2*b*) when judging stimuli corresponding to events in their relative-future as compared with relative-past (irrespective of their self-location in time), in line with previous studies [8,9]. Similar effects were not found in the political domain when comparing performance to left-wing versus right-wing stimuli. For the moral domain, while no significant difference was found between RTs for relative-moral or -immoral stimuli, a significant interaction was found between self-location and self-reference (*F*_1, 27_ = 37.221, *p* < 0.001, $\eta_{P}^{2}$ = 0.580; figure 2*a*). That is, participants responded faster to relative-moral stimuli from their actual moral self-location but responded faster to relative-immoral stimuli while projecting to an immoral self-location. Furthermore, when projecting to an immoral self-location, participants showed higher SRs for relative-immoral stimuli (*F*_1, 27_ = 28.232, *p* < 0.001, $\eta_{P}^{2}$ = 0.511; figure 2*b*).

Comparing the actual blocks of the three studied domains and the lexical control condition revealed a significant difference in RTs between the groups (electronic supplementary material, figure S1; *F*_3,26_ = 3.659, *p* < 0.05). Tukey–Kramer post-hoc tests showed significantly longer RTs for each of the studied domains compared with the control condition (all *p*-values < 0.05). Moreover, participants responded significantly slower to the political domain compared with the temporal and moral domains (both *p*-values < 0.05). These statistical tests are detailed in the electronic supplementary material, tables S1 and S2.

### (b) A distributed brain network, including regions within the precuneus, medial prefrontal cortex, temporoparietal junction, insula and lateral temporal cortex, is active for self-projection across domains

**Figure 3.**
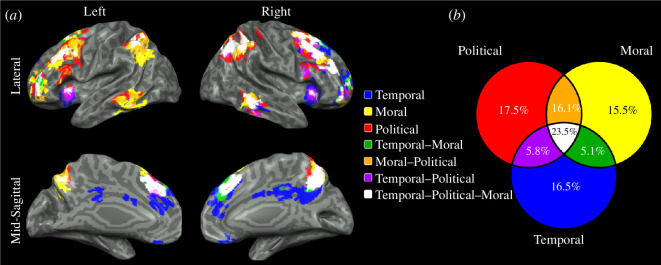
Self-projection-related activations in the temporal, moral and political domains and their intersections. (*a*) Random-effects group analysis of self-projection-related activity, identified by contrasting activity between projected and actual conditions for each domain, showed voxels responsive to self-projection across all three domains in the precuneus, medial prefrontal cortex, dorsolateral prefrontal cortex, temporoparietal junction, and anterior insula, bilaterally and the right lateral temporal cortex (*p* < 0.05, FDR-corrected, cluster size > 25 voxels). (*b*) Group average of the per cent of overlap between active voxels in each domain, demonstrating a partial overlap of 23.5% between all three domains and 27% for two domains.

We first analysed brain activity for self-projection in each of the studied domains (temporal, moral and political). To determine the domain-specific brain regions, we contrasted the activity of the projected condition of each specific domain with the average of the projected condition of the other two domains. This revealed widespread temporal-specific activations in regions within the precuneus, ventromedial prefrontal cortex (vmPFC), angular gyrus, superior frontal cortex and lateral temporal cortex, bilaterally (electronic supplementary material, figure S2: blue). Moral-specific activations included regions within the left dorsolateral prefrontal cortex (dlPFC; electronic supplementary material, figure S2: yellow), while political-specific activations were seen mainly in regions within the right dlPFC and superior frontal cortex (electronic supplementary material, figure S2: red). Next, we determined the extent of domain-general brain regions, that is regions that are involved in at least two domains, by contrasting projected and actual conditions for each domain and assessing for overlapping activations between domains. This revealed voxels responsive to all three domains in regions within the precuneus, mPFC, dlPFC, TPJ and anterior insula, bilaterally, and the right lateral temporal cortex (figure 3*a*). Notably, more than half of the recorded brain activity encompassed more than one domain, with 23.5% of all active voxels active for all three domains and 27% active for two domains (figure 3*b*), emphasizing the implication of the self-projection effect across domains. Domain-specific activity showed a similar extent for each domain—17.5%, 16.5% and 15.5% for the political, temporal and moral domains, respectively (figure 3*b*).

### (c) Predominance of temporal domain-specific brain activity in self-referential processing

**Figure 4.**
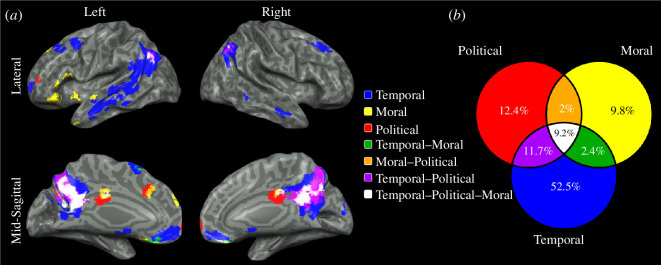
Self-reference-related activations in the temporal, moral and political domains, and their intersections. (*a*) Random-effects group analysis of self-reference-related activity, identified by contrasting activity between each domain and the lexical control task, showed overlapping activations across all three domains in regions within the posterior cingulate cortex, precuneus, ventromedial prefrontal cortex and temporoparietal junction, bilaterally (*p* < 0.05, FDR-corrected, cluster size > 25 voxels). (*b*) Group average of the percent of overlap between active voxels in each domain, demonstrating a partial overlap of 9.2% between all three domains, 16.1% for two domains and 52.5% for the temporal domain exclusively.

We next analysed brain activity for self-reference in each of the studied domains. To determine the domain-specific brain regions, we contrasted the activity of each domain with the average of the other two domains. This revealed widespread temporal-specific activations in regions within the precuneus, vmPFC, TPJ, superior frontal and lateral temporal cortices, bilaterally (electronic supplementary material, figure S3: blue). Moral-specific activations included regions within the left dlPFC, left anterior insula and regions within the mPFC bilaterally (electronic supplementary material, figure S3: yellow), while political-specific activations included regions within the right anterior insula and right dlPFC (electronic supplementary material, figure S3: red). Next, we determined the extent of domain-general brain regions, by contrasting each domain’s activity with a lexical control task and assessing for overlapping activations. This revealed voxels responsive to all three domains in regions within the PCC, precuneus, vmPFC and TPJ, bilaterally (figure 4*a*). Of all active voxels, only 9.2% were active for all three domains and 16.1% for two domains (figure 4*b*). With respect to domain-specific activity, the majority of the overall recorded brain activity (52.5%) was found for the temporal domain, with 12.4% for the political domain and 9.8% for the moral domain, emphasizing the implication of self-reference to MTT.

### (d) Self-projection brain regions mainly overlap with the frontoparietal control network, while self-reference brain regions mainly overlap with the default mode network

**Figure 5.**
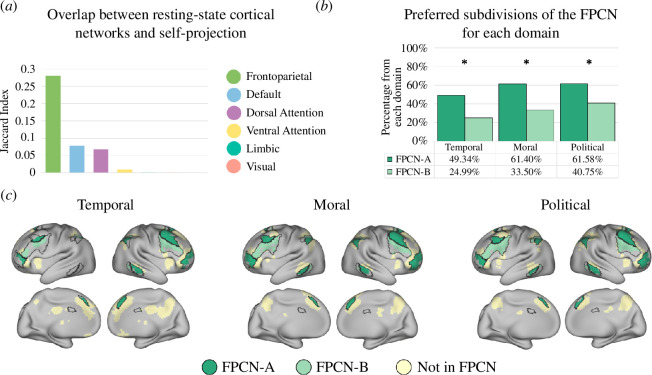
Overlap of self-projection related brain activity with resting-state functional networks. (*a*) Jaccard index (intersection over union) was calculated between the self-projection map and a cortical parcellation to seven large-scale brain networks [17]. The frontoparietal control network (FPCN) exhibited the largest overlap with self-projection-related voxels. (*b*) Distribution of self-projection-related activity for each domain within the subdivisions of the FPCN [17,46]. Percentages represent the proportion of voxels in each subdivision that were activated by each domain. (*c*) Cortical distribution of self-projection-related regions with respect to subdivisions of the FPCN. FPCN regions are outlined in black and are coloured according to each subdivision (FPCN-A: dark green; FPCN-B: light green). Note the similarity between task-evoked and spontaneous (resting-state) brain activity in the FPCN subnetworks.

**Figure 6.**
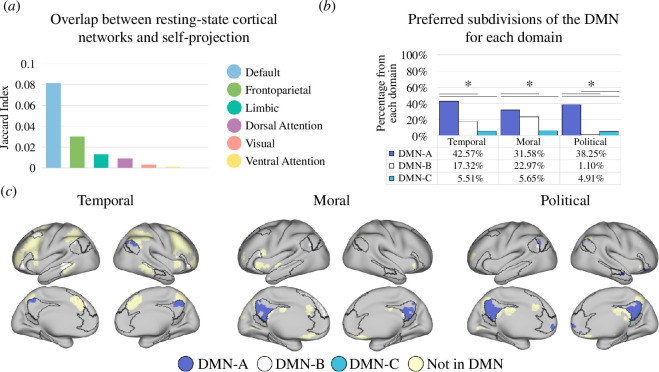
Overlap of self-reference brain activity with resting-state functional networks. (*a*) Jaccard index (intersection over union) was calculated between the self-reference map and a cortical parcellation to seven large-scale brain networks [17]. The DMN exhibited the largest overlap with self-reference-related voxels. (*b*) Distribution of self-reference-related activity for each domain within the subdivisions of the DMN [17]. Percentages represent the proportion of voxels in each subdivision that were activated by each domain. (*c*) Cortical distribution of self-reference-related regions with respect to subdivisions of the DMN. DMN regions are outlined in black and are coloured according to each subdivision (DMN-A: blue; DMN-B: white, DMN-C: turquoise). Note the similarity between task-evoked and spontaneous (resting-state) brain activity in the DMN subnetworks.

To determine the implication of self-projection across domains in spontaneous brain activity, as expressed by the overlap of self-projection-related brain regions with resting-state functional networks, we calculated the Jaccard index (intersection over union) of the self-projection map with a cortical parcellation to seven large-scale brain networks (figure 5*a*) [17]. This revealed that the most dominant similarity to the self-projection map was the frontoparietal control network (0.28), followed by the default mode (0.08) and dorsal attention (0.06) networks, and then the ventral attention (0.001), limbic (0.001) and visual (0.0001) networks. No overlap was found with the somatomotor network. Next, we calculated for each domain the overlap of the self-projection results with two subnetworks of the FPCN and found a significant preference for subnetwork FPCN-A (figure 5*b*). For each domain, the overlap of self-projection regions with three subnetworks of the DMN (3 out of 17 networks) [17] showed preferences for DMN-A and DMN-B (electronic supplementary material, figure S4A,B). Similarly, we calculated the Jaccard index of the self-reference map with the same seven large-scale brain networks (figure 6*a*) [17] and found that the most dominant similarity to the self-reference map was the DMN (0.08), followed by the FPCN (0.03), limbic (0.01), dorsal attention (0.01), visual (0.003) and ventral attention (0.001) networks. No overlap was found with the somatomotor network. Next, for each domain, we calculated the overlap of the self-reference results with three subnetworks of the DMN and found a significant preference for DMN-A for all domains (42.6%, 31.6% and 38.3% for the temporal, moral and political domains, respectively) followed by DNA-B in the temporal (17.3%) and moral (23.0%), but not political (1.1%) domains (figure 6*b,c*). All domains showed a preference for subnetwork FPCN-A, which is functionally related to the DMN (electronic supplementary material, figure S5A) [46]. Task-based functional connectivity analysis revealed co-activation of the PCC (major hub of the DMN-A subnetwork) and the FPCN-A during self-projection in each of the three studied domains, as evident by a higher correlation between the regions during projection time courses compared with actual time courses (electronic supplementary material, table S3; all *p*-values < 0.05). Similarly, a higher correlation between the regions was found for the actual condition compared with the control condition for each domain, indicating coupling of the DMN-A and FPCN-A during self-reference.

## 4. Discussion

Examining functional brain activity during self-projection and self-reference in the temporal, moral and political domains, as well as its comparison to spontaneous brain activity revealed several novel findings. First, we found that self-projection significantly affected RTs and SRs in all three domains, with longer RTs and lower SRs in the projected condition compared with the actual condition. Most of the recorded brain activity during self-projection was found to be domain-general, with 23.5% of the recorded voxels active for all three domains and 27% for two domains, suggesting political and moral self-projection rely on a similar neurocognitive mechanism as does temporal self-projection, a core component of MTT. These domain-general brain regions included the precuneus, mPFC, dlPFC, TPJ and anterior insula bilaterally and the right lateral temporal cortex. Domain-specific brain regions were found to consist of 15.5%–17.5% of all active voxels for each domain. Self-reference in the actual condition of each domain resulted in longer RTs compared with the lexical control condition. Brain activity was dominated by domain-specific activity in the temporal domain (52.5%), in regions including the precuneus, vmPFC, TPJ, superior frontal and lateral temporal cortices, bilaterally. Domain-general self-reference regions consisted of only 9.2% of all active voxels for the three domains and 16.1% for two domains, in brain regions including the PCC, precuneus, vmPFC and TPJ, bilaterally. Domain-general self-projection regions had the highest similarity to the FPCN, followed by the DMN, while self-reference regions showed the opposite pattern with highest similarity to the DMN, followed by the FPCN. Lastly, domain-general self-projection brain regions mainly overlapped with subdivision FPCN-A, while self-reference brain regions showed a preference for subdivision DMN-A for all three domains. Task-based functional connectivity analysis showed co-activation of these networks during self-projection and self-reference. In the following, we discuss the potential significance and implications of our results to the understanding of the complex interplay between temporal, political and moral cognition.

Domain-general and domain-specific brain activities and regions are a fundamental characteristic of the neurocognitive system [50]. The human brain contains a set of functionally general regions enabling cognitive flexibility essential for tackling novel problems, alongside brain regions specialized for specific tasks [51]. The interplay between these two processing modes underpins human cognitive abilities such as language [52], perception [53] and creativity [54]. In recent years, this question has been central in the investigation of self-related cognitive functions as well [55,56]. Specifically, whether the neurocognitive system underlying the ability to self-project in time also supports other forms of self-projection, and to what extent, has been a matter of debate [10,15,57]. The self-projection effect identified here suggests that self-projection for each of the studied domains exerts similar cognitive loads, consistent with prior research [8,9,12,15]. Gauthier and coworkers [14,15,33] investigated the similarities between MTT and mental space navigation by examining participants’ judgements of historical events in time and space from different mental perspectives. Functional neuroimaging results showed that self-projection in space primarily involves the medial and lateral parietal cortices, while self-projection in time engages a widespread frontoparietal network. These two networks overlapped at the right precuneus, inferior parietal lobe and superior frontal cortex [15]. Another study that investigated mental travel in the social domain showed that self-projection involves activity in comparable brain regions, namely the mPFC, precuneus and TPJ bilaterally, the left PCC and inferior frontal lobe, and the right superior frontal lobe [16]. Our findings support the notion of a domain-general system for self-projection, as nearly a quarter (23.5%) of all active voxels were found to be responsive to self-projection in all three domains, and more than a half (53.5%) involved at least two domains, situated in brain regions largely coinciding with these previous studies [15,16]. Taken together, our findings suggest that the ability to self-project is supported by a shared brain system, enabling the imagination of oneself across multiple seemingly distinct domains.

MTT plays a critical role in the evolutionary success of humans [1,4]. Taking this into consideration, and in light of previous results [9,15], we reasoned that the brain system specifically underlying temporal self-projection would involve a widespread functional network in comparison to more confined political- and moral-specific activity. Indeed, our analysis revealed temporal-specific self-projection activity in brain regions within the precuneus, vmPFC, angular gyrus, superior frontal and lateral temporal cortices, bilaterally (while the cortical volume was comparable to the other domains). These regions highly coincide with the ‘core network’ that mediates past and future thinking [20]. Moreover, previous studies found that mPFC subregions have dissociable roles, as self-projection into another person’s perspective recruited the dorsal mPFC [58,59], while as in our study the vmPFC was shown to be involved in temporal self-projection into the personal past [60] and future ([45,46]; but see [47]). Previous works identified that the so-called ‘moral brain’ depends on multiple domain-general contributions, housed in a widespread brain system [61,62]. This encompasses social cognition related regions, particularly involving the right TPJ, which is crucial for understanding others’ mental states during moral judgements [63,64], as well as emotion-related regions, with the vmPFC playing a significant role in integrating emotional and utilitarian aspects of moral judgements [65,66]. According to the dual-process model of moral judgement, the dlPFC supports making utilitarian moral decisions, relying on abstract thinking and the deliberate use of explicit rules [67,68]. In this sense, the left dlPFC, which was found in the current study to be associated with self-projection in the moral domain, may indicate that self-projection to a more immoral perspective drives an abstract-reasoning-based rather than emotional-based moral judgement process [69,70], yet this remains to be tested directly. In the political domain, taking the perspective of one’s own political candidate has been shown to result in increased PCC activation, whereas taking the political opponent’s perspective led to greater activity in regions within the TPJ and insula [71]. These regions (especially the former regions) were found in our study to be part of the domain-general system for self-projection. Nonetheless, our study found that political self-projection elicited domain-specific activations within the right superior frontal cortex as well as the right dlPFC, a region that in the context of politics and neuroimaging has been previously associated with the degree of conservatism in political beliefs [72].

Self-reference encompasses the capacity to refer to different events, political beliefs or moral perspectives, with respect to either the projected or actual self-location (for the self-reference effect in memory see [28–31]). This was measured here as the ordinal relationship between the different stimuli to one’s imagined or actual self-location (past/future, left-wing/right-wing, immoral/moral). Our behavioural results in the temporal domain showed that participants responded quicker and more accurately with respect to future events as compared with past events, comparable to previous studies [8,13]. Note that this effect in the temporal domain is generally more sensitive and less robust than the self-projection effect, as it may be influenced by age, writing direction or clinical states such as spatial neglect [11,12,27]. Nonetheless, studies focusing on the moral and political domains have identified similar effects, suggesting that self-referential processing in these domains is bound to spatial characteristics [73–76], as may be the case also with respect to the temporal domain [11,26,27,77]. For example, the perceived cognitive distance of a political party to one’s own political views had an effect on participants’ reaction times when judging political stimuli [74]. Namely, participants responded faster when acronyms of political parties were presented on the congruent side of the screen (right-wing parties on the right side and left-wing parties on the left side) compared with the incongruent side, an effect which was stronger for right-wing participants when responding to left-wing parties as compared with right-wing parties [74]. In the moral domain, engaging with morally and immorally described avatars modulated the embodied distance to the avatars (closer and more distant, respectively) [75]. Lastly, participants responded faster in the lexical control condition compared with self-reference in the actual condition for all three domains as expected and in alignment with the neuroimaging analysis.

Most of the brain regions active for self-reference were found to be related to the temporal domain (52%), suggesting the importance of self-reference in MTT [12,13,57]. Only 9.2% were found active for self-reference in all three studied domains, including the PCC, vmPFC and TPJ, bilaterally. These regions have been previously implicated in domain-general activity for self-referential tasks in the spatial, temporal and social domains [77–79]. Our results suggest that the self-referential effect engages a shared system to a lesser degree than self-projection, yet comparable to those engaged by such an effect in space, time and person [78]. On the contrary, widespread temporal-specific activations were recorded for self-reference, in line with previous results (e.g. [14]). This result further emphasizes the role of self-reference in MTT, specifically in comparison to the other domains [80]. Alternatively, these widespread activations may be related to the objective chronometrical properties of the temporal domain, compared with the more limited political- and moral-specific activations, which are more subjective and abstract. This is in line with a meta-analysis of 19 studies which found that abstract concepts have a significantly smaller neural footprint, involving only the left inferior frontal gyrus and middle temporal gyrus, compared with concrete concepts, which engaged a much broader network ([79], but see [80]). Furthermore, unlike the political and moral domains, stimuli in the temporal domain may have varied in terms of personal closeness, yet the activation in the vmPFC and absence of rostral anterior cingulate cortex and anterior mPFC activity may suggest that most stimuli consisted of people from distant social scales [81,82]. Finally, with respect to political and moral domain-specific activity, moral-specific activations were found in the left dlPFC, left anterior insula and regions within the mPFC bilaterally, while political-specific activations were found in the right anterior insula and right dlPFC, comparable to the self-projection effect and in agreement with a growing body of research suggests that political and moral cognition are associated with the frontal lobe, specifically the dlPFC [83–85].

In a comparison with resting-state brain networks, self-projection related regions displayed strongest similarity to the FPCN, with the DMN showing the second highest similarity. Conversely, self-reference related regions exhibited the highest similarity to the DMN, while the FPCN showed the second highest similarity. The FPCN (recently taxonomized as L-FPN) [86,87] has been associated with the initiation and flexible adjustment of controlled processes [88] and has been suggested to play a role in integrating externally directed attention and internally directed thought [89]. On the other hand, the DMN is dedicated to a wide range of processes related to self-referential thinking and introspection, including mind-wandering, autobiographical memory retrieval, envisioning the future, social cognition and moral reasoning [34,35,90]. Our findings suggest self-projection and self-reference initiate an interplay between the DMN and FPCN. Flexible coupling between the two networks was shown to support internally focussed, goal-directed cognitive processes like autobiographical planning or mentalizing based on linguistic information, suggesting that other introspective processes requiring simultaneous goal-directed control of information may involve both networks [91–94]. Likewise, a functional interaction in theta band between the DMN and FPCN-A, a subdivision of the FPCN [46], was shown to support task-relevant internally directed attention [95]. In this sense, the act of self-projecting into a distinct temporal, moral or political self-perspective and sustaining this self-projection throughout the task may have necessitated an interaction between the FPCN-A subnetwork and the DMN. This is supported by the positive correlation found between the PCC hub of the DMN-A and FPCN-A activity during self-projection in all domains. Similarly, co-activation of these networks was found during self-reference. Moreover, examining the overlap of self-reference related brain regions with three subdivisions of the DMN showed a preference for DMN-A for all three domains, consistent with its role in mediating self-referential cognitive processes alongside processes that require both memory and social cognition [18]. While self-projection brain regions overlapped with DMN-A as well, they showed a similar preference to DMN-B, a network that has been related to social cognition and ToM [18]. Taken together, our study highlights the intricate involvement of specific DMN and FPCN subnetworks in self-referential and self-projection processes, respectively. This reinforces the idea that MTT is not a standalone cognitive system [1]. Rather, it consists of multiple underlying mechanisms that may share neural substrates with analogous cognitive functions in other domains. Conversely, self-referential activity was related mostly to time, unlike ‘travels’ in the other domains.

Our study is subject to several possible limitations. Foremost, while political and moral views are elaborate and complex, in this study, we opted for the use of people’s names which serve only as a proxy for these ideas. Nonetheless, this allowed the representation of complex abstract stimuli in a reduced form which is both suitable for an fMRI paradigm and importantly enables unified stimuli across the experimental conditions. The lexical control task used here controls most aspects of the task, yet participants are not necessarily required to mentalize or recruit episodic memory to successfully perform the task. Therefore, our analyses also included comparisons between domains over the same task, which are discussed here as the identified domain-specific regions. Moreover, including self-projection in multiple directions (such as future and past, moral and immoral, conservative and liberal) would have been beneficial, but the large number of conditions associated with such an approach would have posed practical challenges, such as requiring a large number of repetitions unsuitable for a single experimental session. For similar reasons, a control condition requiring participants to self-project in a separate orthogonal domain while performing the task in the studied domains was not included in the experimental design, although this could have enhanced the internal validity of the behavioural results. Finally, our results may be influenced by the right-left answers owing to a space associated response code, especially with respect to the political condition. Yet, we have associated the left-right responses with the common directionality for each domain, and subjects were all from the same culture, thus minimizing a potential between-subject effect. Response code effects and potential influences in the political and moral domains should be investigated in further studies, in search for effects comparable to the SNARC and the STEARC (Space-Number and Spatial-Temporal Association of Response Codes, respectively) effects [96–98].

In conclusion, this study demonstrated that self-projection to different moral and political perspectives engages a shared brain system similar to that involved in MTT, raising the possibility that the brain may flexibly apply subprocesses of MTT to facilitate higher-order cognitive functions. Conversely, self-reference was found to involve mostly the temporal domain. MTT may thus be regarded as a ‘mental-experiential travel’, with self-projection as a domain-general construct and self-reference as domain-specific.

## Data Availability

Data is available in the electronic supplementary material [99]. Data and analysis code are also available at [100].
